# Survival of people on antiretroviral treatment in Zambia: a retrospective cohort analysis of HIV clients on ART

**DOI:** 10.11604/pamj.2016.24.144.6004

**Published:** 2016-06-15

**Authors:** Patrick Amanzi, Charles Michelo, Christopher Simoonga, Rosalia Dambe, Gershom Chongwe

**Affiliations:** 1 University of Zambia, School of Medicine, Department of Public Health; 2 Ministry of health, Zambia

**Keywords:** Survival analysis, retrospective cohort, lost to follow up

## Abstract

**Introduction:**

Provision of free anti-retroviral therapy in Zambia started in June 2004. There were only 15,000 people on treatment as at December that year, mainly due to lack of access. This number rose to 580,000 people as at December 2013. The general objective of this study was to determine survival of people on ART and to examine associated predictors for survival.

**Methods:**

The study included ART patients enrolled between the year 2002 and 2013 (n=10,395) in 285 health facilities in Zambia. Patient files were analyzed retrospectively. The study used Kaplan Meier and Cox-proportional hazard models to describe the relationship between lost to follow up and age, sex, baseline CD4 cell count and weight.

**Results:**

Results showed that lost to follow up accounted for 90% of the clients that had dropped out, while 10% was to deaths. Low baseline CD4 count (p-value 0.001, HR 0.9994, (95% CI 0.9993, 0.9996) at initiation was associated with lost to follow up together with weight at initiation (p-value 0.031, HR 0.9987 at 95% CI (0.9975, 0.9998)) of ART.

**Conclusion:**

This study has demonstrated that lost to follow up is a substantial contributing factor to drop outs among HIV patients on treatment. Strengthening of community treatment supporters especially immediate family members in emphasizing to the client the need to continue treatment is necessary. The health facility could do more in emphasizing the importance of treatment especially in the initial stages. Further, in order to reduce opportunistic infections and probable deaths during treatment, cotrimoxazole prophylaxis should be maintained so as to raise the CD4 levels. Improved nutritional assessment and counseling to boost the nutritional status of the clients throughout should be encouraged.

## Introduction

The beginning of Anti-Retroviral Treatment (ART) in 1996 led to a revolution in the care of patients with AIDS in the developed world. Although this treatment does not cure and also presents new challenges with respect to side effects and drug resistance, it has dramatically reduced rates of morbidity and mortality, has also improved the quality of life of people with HIV/AIDS and has revitalized communities [1]. Zambia has one of the most devastating HIV and AIDS epidemics. Since the first reported case of HIV in the country in the year 2004, Zambia has seen high rates of prevalence with 15.6 in 2002 and 14.3 in 2007 [2]. Despite receiving millions of dollars for HIV programs over the years, the overall prevalence of HIV remains high, with some areas such as Lusaka posting over 19% [3]. In the year 2002, the Zambian government took a policy decision to make ART available to everyone needing treatment and allocated 3 million US dollars to purchase ARV drugs for 10,000 people, to be provided through the public service [4]. The provision of ART was recognized as an integral component of the multi-sectoral response to HIV/AIDS. This was provided on a cost-sharing basis. In June 2004, the Ministry of Health (MoH) started providing free treatment to people eligible for ART. Since then, the ART program has been scaled up from the two initial pilot hospitals to over 550 of the 2000 public and private health facilities across the country [5, 6]. The new policy includes free drugs, basic laboratory tests and CD4 counts. Over 80% of those eligible for ART are actually on it [5] and focus has shifted to maintaining these people on treatment thereby increasing the rate of survival on treatment. It is estimated that in Sub-Saharan Africa between 8% and 26% of patients die in the first year of ART, with most deaths occurring in the first few months [7]. Mortality rates are likely to depend not only on the care delivered by ART program, but more fundamentally on how advanced disease is at program enrolment and the quality of preceding health-care According to the Zambian MoH, only 81% of people commenced on ART survived a full year on treatment in 2013[5], and the number is even lower at longer duration of follow up, i.e. 24, 36 months and beyond. Among the factors identified as contributing to this are low basal metabolic index (BMI) at commencement of ART as well as late presentation of patients to the ART clinic. This study is aimed at assessing the survival of patients on the program in the sampled facilities and also whether there was a shift at presentation at initiation of treatment from late to early.

## Methods

**Population and sampling procedures**: The data used in this study stems from records for all clients ever enrolled (n=10,395) in 285 ART facilities from 2002 to 2013 which is stored in an electronic clinical database called SmartCare. Smart Care is a patient level electronic system meant to enhance the quality of care to HIV and other diseases and conditions. It collects variables such as, but not limited to, sex, date of birth, CD4 count at commencement of ARVs, registration date and status of patient (either dead, lost to follow up (LTFU), transfer out and alive) at a particular time. To examine survival rates and associated predictors, a retrospective cohort design was used. Records for all clients ever enrolled in the 285 ART sites in the country were analyzed from 2002 up to December 2013 retrospectively. Enrolment dates and current status were recorded. Time intervals at which these events occurred were recorded as they were essential to the analyses. A patient is declared LTFU when they miss an appointment and efforts to trace the client for 90 days have proved futile. A patient is declared dead if they die in the hospital and the information reaches the ART clinic or if during the follow up time, the ART clinic has been informed that its client died in the hospital or community without the information reaching the ART clinic at the time of occurrence. The study used all ART sites in Zambia which had a fully operational Smart Care system for patient care and had all been operational as ART sites for at least 36 months Prior check up with Ministry of Health Zambia indicated that 285 facilities met the requirement for this research.

**Data extraction**: From the 285 facilities, about 25,000 patient records entered in Smart Care were reviewed. All patient records with complete records entered were eligible to be reviewed. The main criteria for the inclusion into the study were any HIV positive client who subsequently commences ART at a particular health center.

**Data analysis**: Analysis and interpretation of data was done with the help of; STATA version 12 [8]. We used survival analysis at intervals 12, 24, and 36 months and period beyond to study the survival of clients on the program. Variables such as age, baseline CD4 count, and sex were analyzed with variable LTFU (main outcome variable) to determine associations. The Kaplan-Meier estimates [9] were used to estimate the survival probability (of LTFU) after ART initiation, and p values to compare the curves of survival. The Cox proportional hazard model was used to assess the relationship between baseline variables with LTFU. The study received approval from Excellence in Research Ethics and Science (ERES) converge ethics committee and the Ministry of Health Zambia.

## Results

**Sample characteristics**: Overall, 34.8% of the patients were male and 65.2% were females. Just over nine percent were children below the age of 14 years and 90.7% were adults 15 years and above. The average age of the clients was 31 (SD±13.3) years with the minimum age of 0 and maximum of 85 years.

**Antiretroviral treatment**: Although the first client was enrolled in 2002 at University Teaching Hospital (UTH), the majority were enrolled from 2008 onwards. There was a significant association between the year a patient was enrolled on the program and whether or not they were part of the study at the end of 2013 (p-value < 0.001). For ART retention, results show that from 2002 to December 2013, the program was only able to retain 42% of the clients ever enrolled on the ART program. Over 90% of the patients that had dropped out of the ART program by the end of 2013 were due to LTFU, 8.8% through confirmed death and only 0.57% were in default at the time of data collection. Overall, from the first person enrolled in 2002 to 2013, 64% of males and 53% of females were either LTFU, dead or in default but the difference were found not to be statistically significant (p- value = 0.12, 95% CI (0.9994, 1.0044)). Table 1 below illustrates the percentage drop out of patients on the ART program over the 11 year period. The highest dropout percentage was observed in the year 2006. As one would expect, there is a decline as you approach the final year of 2013 since the more recent one is enrolled on the ART program, the less the chances of dropping out of the program. The increase from 2002 to 2006 may be attributed to the fact that there were few numbers of clients enrolled at that time as compared to the latter years. Table 1 also shows that the ART program lost over 37% of its clients due to LTFU and deaths within the first 12 months, and lost as many as 46% and above 50% of clients after 24 months and 36 months respectively. Intuitively, the only patient enrolled in the program in the year 2002 was not present in the program 11 years on. As many as 73% of the total clients enrolled in 2006 and sampled 2002 had dropped out of the program in the year 2013. This is taking into consideration the higher numbers of enrolment in the years between 2006 and 2013 due to an increased number of facilities offering ART. In the end, about 58% of clients ever enrolled in the selected facilities had dropped out of the program. The differences between year of registration and whether one is lost to follow up or not at the end of 2013 were found to be significant with p-value less than 0.0001 at 95% confidence level.

**Table 1:** Percentage drop out of ART patient by year of enrolment

Year of registration	Total number of ART clients enrolled	Total number of drop outs(LTFU &deaths)	Percentage dropout
2002	1	1	100%
2003	5	2	40%
2004	121	76	63%
2005	383	251	66%
2006	638	465	73%
2007	958	682	71%
2008	1,522	1,036	68%
2009	1,574	941	60%
2010	1,657	821	50%
2011	1,302	755	58%
2012	1,310	606	46%
2013	924	344	37%
Total	10,395	5,980	58%

**Figure 1:**
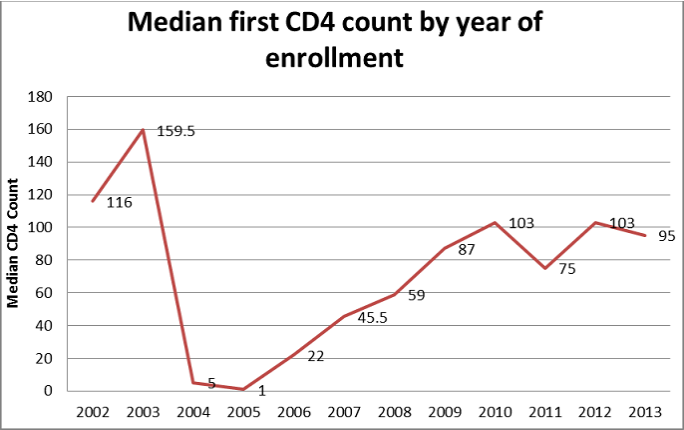
Median CD4 count distribution by year of enrollment

**Figure 2:**
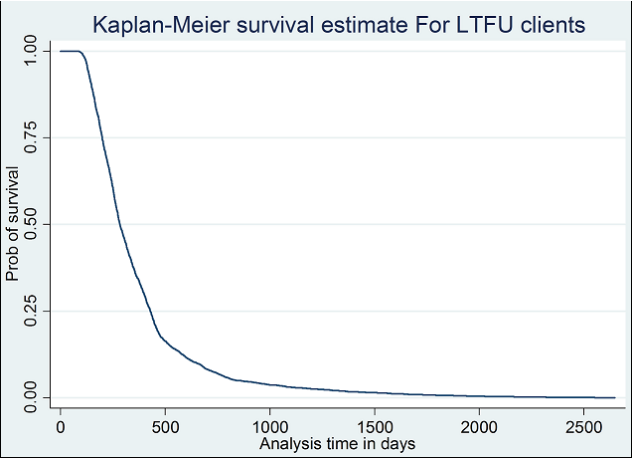
Survival estimate for LTFU clients

**Figure 3:**
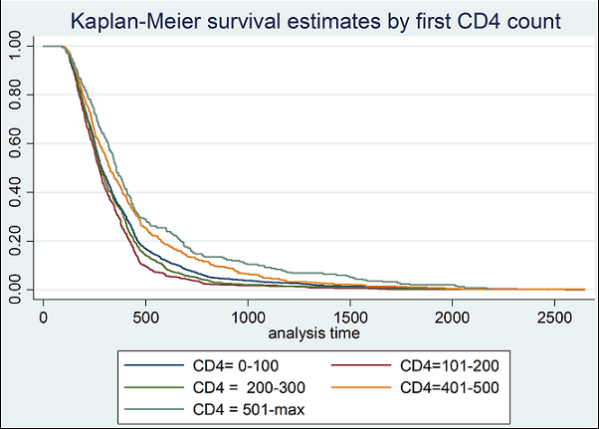
Survival estimate for LTFU clients by baseline CD4 count

**Determinants of survival and mortality**: About 70% of the patients had their baseline CD4 count taken at enrollment into the ART program. Figure 1 below shows the median CD4 count distribution over the years at enrollment into the ART program. As shown in Figure 1, the median CD4 count increased from as low as 22 cells/μl in 2006, to about 95cells/μl in 2013, peaking at 103 cells/μl in the years 2010 and 2012. An analysis was conducted to compare the median CD4 count during period 2006 to 2010, the period in which Zambia was using the 2006 ART guidelines that recommended commencing ART at CD4 200 cells/μl and below with period 2011 to 2013 in which the country was using the 2010 guidelines that recommended commencing ART at CD4 350 cells/μl and below. The mean CD4 at commencement of ART was 173 cells/μl and 191 cells/μl for the two periods in question respectively. The finding however was found not to be statistically significant. A multivariate analysis (Cox-Proportional Hazard Model) was conducted on several variables to determine their effect on LTFU. Table 2 below shows the model taking into consideration age, sex, baseline CD4 count, weight at registration of patient and year of patient registration. Results in the table indicate that the patient's age taking into consideration, sex, baseline CD4 count and weight at registration of patient had no effect on the patient being LTFU at the end of study (p-value of 0.12). Similarly, sex of client was found not to have an effect on LTFU (p-value = 0.23) taking into consideration age, baseline CD4 count, weight and year of registration. Baseline CD4 count and year of registration were found to have an effect on LTFU of ART clients (p-value < 0.001). The patient's weight at baseline was also found to have an association with LTFU (p-value = 0.031). For the patients that were LTFU, the average length they stayed on treatment was 366 days (1 year) with the least being 90 days and the longest to have stayed on treatment was 2647 days (over 7 years). The total time at risk of LTFU was 1,799,676 person days. The study also analyzed the probability of survival using the Kaplan-Meier survival curve. The results are shown in Figure 2 below. The figure indicates that the probability to survive in the first 90 days was almost one but steeply drops. The probability to survive beyond 500 days (approximately 1 and half years) is below 0.25 and stabilizes thereafter at about 0.1 and eventually zero beyond the 2500 day's period Further analysis was done to compare baseline CD4 count and the probability of survival for the clients that were LTFU. Figure 3 below represents this information broken down by the category of CD4 cell count. Figure 3 indicates the relationship between baseline CD4 count and the probability of survival for the clients that were LTFU. CD4 counts between 0-100 cells/μl and 101-200 cells/μl were associated with lower probabilities of survival compared with higher CD4 levels. A life table was drawn showing probability of survival at intervals of 90 days (3 months). As expected, there was a higher probability of remaining in the study for the people that were LTFU; at 3 months probability. 0.99 with 95% CI (0.9986, 1), probability of 0.8132 at 6 months with 95% CI (0.802, 0.82), to as low as probability of 0.0004 at 2520 days (7 years) of being on treatment with 95% CI (0.0001, 0.001) the information is summarized in Table 3 below showing the life table for the people that were LTFU.

**Table 2:** Effect of age, sex, baseline CD4 count, weight at enrollment and year of registration on LTFU using Cox-Proportional Hazard Model

	Hazard Ratio	P-value	95%C.I.
Age	1.0019	0.12	0.9994- 1.0044
Sex	0.9632	0.23	0.9057- 1.0244
Baseline CD4 count (cells/μl)	0.9994	0.001	0.9993- 0.9996
Baseline Weight	0.9987	0.031	0.9975, 0.9998
Year of registration	1.3647	0.001	1.3402-1.3897

**Table 3:** Life table indicating for probability of remaining in the study for patients that were lost to follow up from enrolment on ART

Interval (days)		Beg. Total # of clients	LTFU	Probability of remaining in the study	95% CI
[0	>90]	4908	1	0.9998	0.9986- 1
[90	>180]	4907	916	0.8132	0.802-0.82
[180	>270]	3991	1333	0.5416	0.5275-0.55
[270	>360]	2658	913	0.3555	0.3422-0.36
[360	>450]	1745	704	0.2121	0.2008- 0.22
[450	>540]	1041	342	0.1424	0.1328-0.15
[540	>630]	699	182	0.1053	0.0969-0.11
[630	>720]	517	137	0.0774	0.0702- 0.08
[720	>810]	380	109	0.0552	0.0491-0.06
[810	>900]	271	45	0.046	0.0404- 0.05
[900	>990]	226	41	0.0377	0.0326- 0.04
[990	>1080]	185	30	0.0316	0.027- 0.03
[1080	>1170]	155	20	0.0275	0.0232- 0.03
[1170	>1260]	135	20	0.0234	0.0195- 0.02
[1260	>1350]	115	20	0.0194	0.0158- 0.02
[1350	>1440]	95	17	0.0159	0.0127- 0.01
[1440	>1530]	78	10	0.0139	0.0109-0.01
[1530	>1620]	68	12	0.0114	0.0087- 0.01
[1620	>1710]	56	12	0.009	0.0066- 0.01
[1710	>1800]	44	9	0.0071	0.0051- 0.009
[1800	>1890]	35	6	0.0059	0.0041- 0.008
[1890	>1980]	29	8	0.0043	0.0027- 0.006
[1980	>2070]	21	5	0.0033	0.002- 0.005
[2070	>2160]	16	2	0.0029	0.0017- 0.004
[2160	>2250]	14	5	0.0018	0.0009- 0.003
[2250	>2340]	9	1	0.0016	0.0008-0.003
[2340	>2430]	8	1	0.0014	0.0007- 0.002
[2430	>2520]	7	3	0.0008	0.0003- 0.002
[2520	>2610]	4	2	0.0004	0.0001- 0.001
[2610	.	2	2	0	-

## Discussion

This 11 year retrospective cohort survival analysis gives insights into the length of time the ART clients stay on treatment. The average age of clients was found to be 31 years which compares well with similar studies across Africa. A similar study done in Ethiopia had a mean of 33 years [10] and another done in Cameroun had mean of 31 years [11]. The earliest patient was enrolled in 2002 and the latest in 2013. As expected, the earlier one is initiated, the less chances they had of being alive and on treatment in 2013. The cumulative dropout was 58% as the program could only maintain 42% of clients in the study. This may compare well with a 5 year retrospective cohort study done in India which reported a cumulative dropout of 37.7% [12]. The LTFU was found to account for 90% of the cumulative drop outs in this study. Further comparison with the factors associated with LTFU found similar results. Having a CD4 count less than 100 cells/μl was found to be associated with LTFU in this study and this was consistent with the results found in the 5 year cohort India study which also had CD4 <100 cells/μl associated with LTFU [12]. The cumulative deaths for this study were found to be 10%, consistent with a 5 year cohort study done in Ethiopia which also had a cumulative deaths at 10.4% [13], although other studies done in other African settings posted much higher deaths, i.e 29.7% in a study in Tanzania [14] and 23% in Cameroun [11]. This could be attributed to the fact that most of the deaths could have occurred unregistered by the program in Zambia. A large part of the LTFU in this study may have died but the program did not have that information hence the clients were classified as such. One of the major findings for Sample Vital Registration on Verbal autopsy [15], a study on the causes of community deaths in Zambia revealed that 20% of community deaths were due to HIV. It is thus expected that the large number of the LTFU clients may have died in the community without the information reaching the respective ART clinic of a health center. This study did not follow up the clients in the community to establish if they had died or not.

The low rates of survival seen in the clients in the first year of being on ART as shown in the life table (Table 3 below) could easily be attributed to community deaths. This compares well with high mortality in other studies 29.7% and 23% in Tanzania [14] and Cameroun [11] respectively occurring in the first 12 months post commencement of ART. The two studies actually followed up the deaths in the communities which this study did not do. The Kaplan-Meier estimate also confirms these results as shown in Figure 2 below. In comparison, sex and age were found to have no significant bearing on whether the patient is LTFU or not. This finding is also similar to other studies in African patients. A study in Ethiopia also found no correlation between sex and the chances of being LTFU [10]. The striking finding was the relationship with CD4 count at the time of presentation. The lower the CD4 count at initiation, the higher the chances of LTFU and the relationship was found to be significant. For those that had CD4 count less than 200 cells/μl, their chances of survival were lower than the category with CD4 count above 200 cells/μl as shown in Figure 2 (Survival estimate for LTFU clients by baseline CD4 count). This may confirm that most of the patients deemed LTFU may have actually died out in the community without the health center in question having the information. This finding is consistent with studies across the world, more strikingly studies in the African setting of Tanzania [14] and Cameroun [11] and Ethiopia [13].

With a change of protocol by WHO of commencing people on treatment from CD4 count below 200 cells/μl [16] to commencing on treatment below 350 cells/μl in the 2010 guidelines [17], one would expect a huge leap of median CD4 cell count at commencement of ART. As seen in Figure 1 median CD4 count at initiation of ART has remained worryingly low, moving from 22 cells/μl in 2006 to 95 cells/μl in 2013 with highs of 103 cells/μlin 2010 and 2012. This is despite the threshold changing from commencing ART in people with CD4 below 200cells/μl as per 2006 guidelines to about CD4 350 cells/μl in the 2010 ART guidelines. With CD4 being one of the major contributors of LTFU, the lack of increase in the median poses some major challenges to the ART program. Weight at initiation was also critical as observed in the results above. The lower the weight at initiation intuitively may suggest that the client is weaker and sicker. As it increases the chances of LTFU, it also increases the chances that client dies off in the community or in other departments of the hospital without ART clinic having the information. As observed above, these results are consistent with program data that suggest that 20% of clients are lost from the program within 12 months of initiation [18]. The findings from the study suggest the Ministry of Health lose an even higher number of clients at 37% in the first 12 months of initiation than the earlier suggested 20%. This result was consistent with a cohort study done in Ethiopia which posted as high as 38.6% LTFU within the first 12 months[13]. The ministry further loses 46% and above 50% in 24 and 36 months respectively. A sample vital registration with verbal autopsy (SAVVY) conducted in Zambia (2010-2012) showed that over 20% of community deaths are due to HIV [15]. This can also support the study assertions that the majority of the people that are LTFU end up dead without the information reaching the health facility. The study indicated that about 10% of the clients had died. The cause of deaths was not studied in this study. The strength of this study is due to the fact that it covers the whole country with diverse scope in terms of the health centers involved. All levels of health care were part of the sample and thus can be replicated with a larger sample within the country and in other countries as well. Some weaknesses of the study included lack of more background characteristics of the patients such as economic status to link to the possible cause of low BMI and baseline CD4 count at initiation of therapy. It also did not include the interviews with the patients currently on ART to determine some challenges they may be facing while taking medication which may lead to LTFU.

## Conclusion

The study has shown high rates of LTFU within the first 12 months of initiation antiretroviral of therapy in Zambia. Other studies on the same had observed high mortality in the same period thus, the majority could have died off. The rate was particularly high for those that had low CD4 count at commencement of ARVs. This was also true for those that had low weight at commencement of ART. With the median CD4 count remaining relatively low despite increasing the baseline CD4 count from 200 cells/μl to 350 cells/μl in the 2006 and 2010 respectively, more sensitization still needs to be done to actually make sure clients present early for commencement of treatment. Early diagnosis and initiation of treatment is key to the long term success of the ART program. With the high rates of LTFU, It is further recommended that the network of ART adherence supporters be supported with resources and training to enable them carry out the follow up effectively. In order to reduce opportunistic infections and probable deaths, cotrimoxazole prophylaxis should be given to all HIV positive clients as per WHO recommendation. The relationship of weight and LTFU established by the study would require vigorous nutritional assessment and counseling for all HIV positive patients in order to boost the nutritional status of the clients at the beginning and during the course of taking medication.

### What is known about this topic

- Most lost to follow up occurs to the patients with CD4 count below 200 cells/μL in other countries;
- Best practice is to commence ART in CD4 200 cells/μL bringing net benefit of 14.5 life years;
- About 28% of the clients are lost to follow-up within the first 12 months.

### What this study adds

- Lost to follow up was at 37% within the first 12 months of therapy;
- Median CD4 count at enrolment of ART improved from as low as 22cells/μL in 2006, to about 95cells/μL in 2013 with as high as 103 in the years 2010 and 2012. This is in line with the progression of protocols over the years;
- Low baseline CD4 count (p-value 0.001, HR 0.9994, (95% CI 0.9993, 0.9996)) at initiation was associated with lost to follow up of clients on ART.
